# Chest L-Transformer: Local Features With Position Attention for Weakly Supervised Chest Radiograph Segmentation and Classification

**DOI:** 10.3389/fmed.2022.923456

**Published:** 2022-06-02

**Authors:** Hong Gu, Hongyu Wang, Pan Qin, Jia Wang

**Affiliations:** ^1^Faculty of Electronic Information and Electrical Engineering, Dalian University of Technology, Dalian, China; ^2^Department of Surgery, The Second Hospital of Dalian Medical University, Dalian, China

**Keywords:** weakly supervised, lesion segmentation, transformer, local feature, chest radiograph

## Abstract

We consider the problem of weakly supervised segmentation on chest radiographs. The chest radiograph is the most common means of screening and diagnosing thoracic diseases. Weakly supervised deep learning models have gained increasing popularity in medical image segmentation. However, these models are not suitable for the critical characteristics presented in chest radiographs: the global symmetry of chest radiographs and dependencies between lesions and their positions. These models extract global features from the whole image to make the image-level decision. The global symmetry can lead these models to misclassification of symmetrical positions of the lesions. Thoracic diseases often have special disease prone areas in chest radiographs. There is a relationship between the lesions and their positions. In this study, we propose a weakly supervised model, called Chest L-Transformer, to take these characteristics into account. Chest L-Transformer classifies an image based on local features to avoid the misclassification caused by the global symmetry. Moreover, associated with Transformer attention mechanism, Chest L-Transformer models the dependencies between the lesions and their positions and pays more attention to the disease prone areas. Chest L-Transformer is only trained with image-level annotations for lesion segmentation. Thus, Log-Sum-Exp voting and its variant are proposed to unify the pixel-level prediction with the image-level prediction. We demonstrate a significant segmentation performance improvement over the current state-of-the-art while achieving competitive classification performance.

## 1. Introduction

The chest radiograph is widely applied for the diagnosis of thoracic diseases. Diagnostic imaging often requires the classification of findings, as well as their geometrical information. Segmentation of lesions is an indispensable part of clinical diagnosis (1). Deep learning models have achieved considerable success in chest radiograph segmentation (2–4). Unfortunately, these supervised models require substantial pixel-level annotated data to locate the lesions (3–5). The pixel-level annotated medical data are prohibitively expensive to acquire with long working hours of expert radiologists. On the contrary, image-level annotations can be relatively easy to access with the text analysis techniques on radiological reports (6, 7). Thus, a good alternative to supervised learning is weakly supervised learning, which leverages image-level annotations to search the segmentation prediction (8). Existing deep learning models for weakly supervised medical segmentation class the images with features extracted with convolutions (9–12). The pixel-level and image-level predictions are unified with algorithms based on Multiple-instance learning (MIL) (9, 10, 13) or class activation map (CAM) (11, 12, 14). Moreover, the attention mechanism is adopted to promote their performances (9–12). However, these weakly supervised models do not consider the critical characteristics of chest radiographs: the global symmetry of lungs and dependencies between lesions and their positions.

There is an imperfect symmetry between the left and right lungs (15), which the existing weakly supervised models don't take into account. They extract global features from the whole image and it is unclear how the latent feature space is related to the pixel space (9–12). The global symmetry of the lungs can lead these models to contrast symmetrical positions in the left and right lungs to classify the lesions (9). As a result, features of lesions appear at the symmetrical positions of the lesions in the feature space, and the symmetrical positions are misclassified as lesions (9).

Convolutional neural networks (CNNs) with restricted receptive fields have been applied to relate the feature space and the pixel space exactly (16–18). In these models, the images are sliced into patches and the features are extracted within local patches (16–18). The class evidences produced by local features are averaged across all patches to infer the image-level labels with the softmax activation (16–18). However, the selection of the patch size is a hard problem for CNNs with restricted receptive fields to apply in weakly supervised segmentation. Increasing the patch size expands the receptive field and leads to better local features for classification, but coarsens the segmentation output (16–18). Another problem is the way to aggregate pixel-level evidences to the image-level decision. Unlike the images used in (16–18), all of which contact objects, the medical image datasets contain an extra class: no lesion. Averaging the class evidences, patches have the same weight to infer the image-level class. In the abnormal images, the patches with no lesion are more than those with lesions. To assign the right label to the images with lesions, many patches with no lesion may be classed as lesions. The patch with more evidence of lesion should have larger weights in the aggregation. There is another common function for aggregation: the max function, which encourages the model to just consider the most-likely lesion patch (13). But training with just one patch of the whole image, the model is hard to converge (19). Moreover, chest radiographs contain special areas, like the muscle and the black background, which are unrelated to thoracic diseases. It is necessary to filter them out in the aggregation of patches. Moreover, the softmax activation is designed for mutual exclusion. But different diseases can appear in one chest radiograph and may even have an overlapping region.

Another characteristic of chest radiographs is the dependencies between lesions and their positions. Thoracic diseases often have special disease prone areas in chest radiographs. This fact implies a relationship between the lesions and their positions. Weakly supervised deep learning models highlight salient parts of feature maps and separate redundant information with CNN attention modules to promote their performance (9–12). These CNN attention modules treat areas of the whole image equally, with the same convolution and pooling operations (9–12). But the salient parts are more likely located in the disease prone areas and extra attention should be paid to these areas. These models lack the ability to model the position information present in chest radiographs.

To tackle the aforementioned problems, we propose a weakly supervised deep learning model, called Chest L-Transformer, for lesion segmentation and disease classification on chest radiographs. Chest L-Transformer completes these two tasks only using image-level annotations. We present a new restricted receptive field CNN, called Restricted ResNeXt, as the backbone of Chest L-Transformer. Restricted ResNeXt extracts local features with a restricted receptive field and relates the feature space and the pixel space exactly. Hence, the features of lesions only appear at nearby positions of themselves, and the misclassification caused by the symmetry is avoided. Furthermore, Restricted ResNeXt extracts the local features not only from image patches but also from a limited nearby area around them. It can expand the receptive field while maintaining the fine scale of the segmentation output. A particular voting function, called Log-Sum-Exp voting, is proposed to aggregate pixel-level evidences. With this function, patches with differential evidences will have different weights to infer the image-level classes. Furthermore, a variant of Log-Sum-Exp voting is proposed to filter the unrelated areas. To ensure that multiple diseases can be detected simultaneously, the sigmoid activation takes place of the softmax one. Finally, Transformer attention mechanism (20) is introduced into the attention block of Chest L-Transformer to utilize the dependencies between the lesions and their positions. The attention block focuses on the disease prone areas with additional learnable positional embeddings (20, 21). We demonstrate a significant segmentation performance improvement over the current state of the art with competitive classification performance.

## 2. Methods

**Figure 1:**
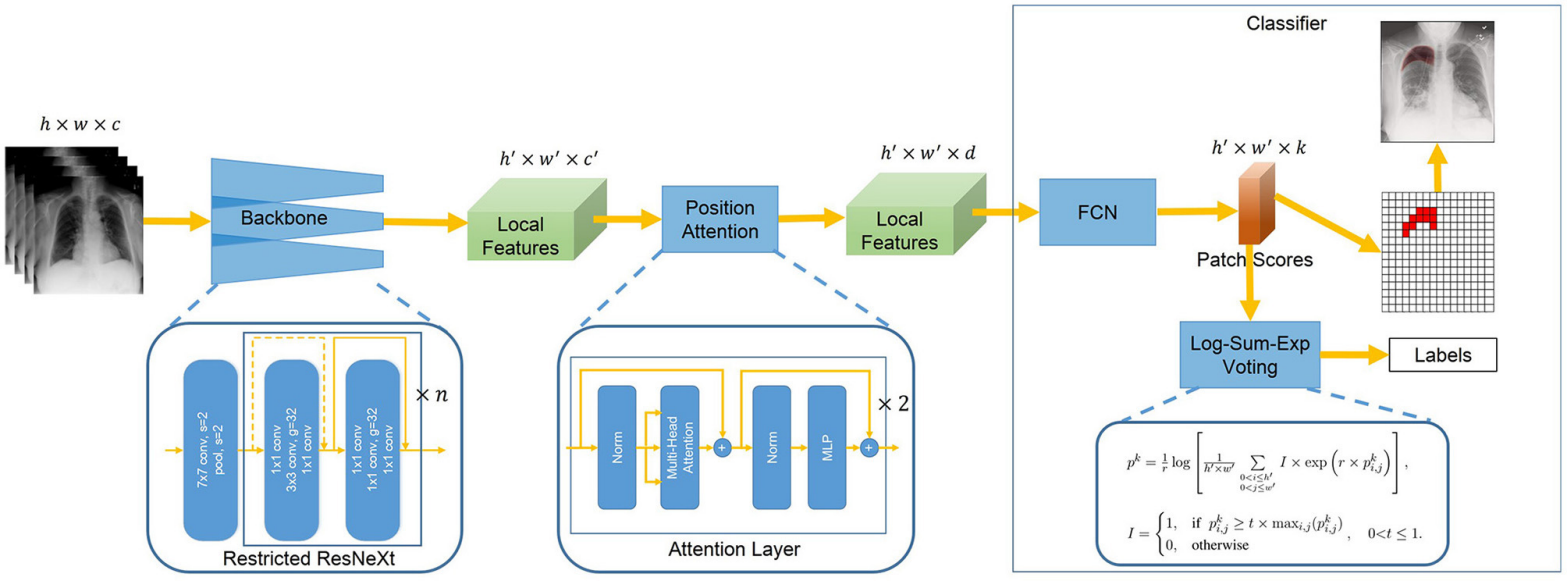
Overview of Chest L-Transformer. A backbone with restricted receptive field CNNs is utilized to extract the local features. The position attention is added to Chest L-Transformer to highlight the features of the region of interest with positional embeddings. The local features are passed to a classifier for the prediction of the image labels as well as the lesion segmentation.

With image-level annotated images, we aim to design a deep learning model that simultaneously produces disease classification and lesion segmentation. The proposed architecture is shown in Figure 1. It consists of three components: backbone, position attention block, and classifier. The backbone extracts the local features with Restricted ResNeXt. The local feature maps are downsampled and each pixel of the feature maps represents a small patch in the original image. The features of the region of interest are highlighted by the position attention block, which is mainly realized by two attention layers. The classifier first assigns each patch a probability of the lesion for the segmentation task by the fully convolutional network (FCN). Then, Log-Sum-Exp voting allocating patches with differential evidences differential weights are used by the classifier in inferring the image-level classes with the probabilities of patches.

### 2.1. Backbone

**Figure 2:**
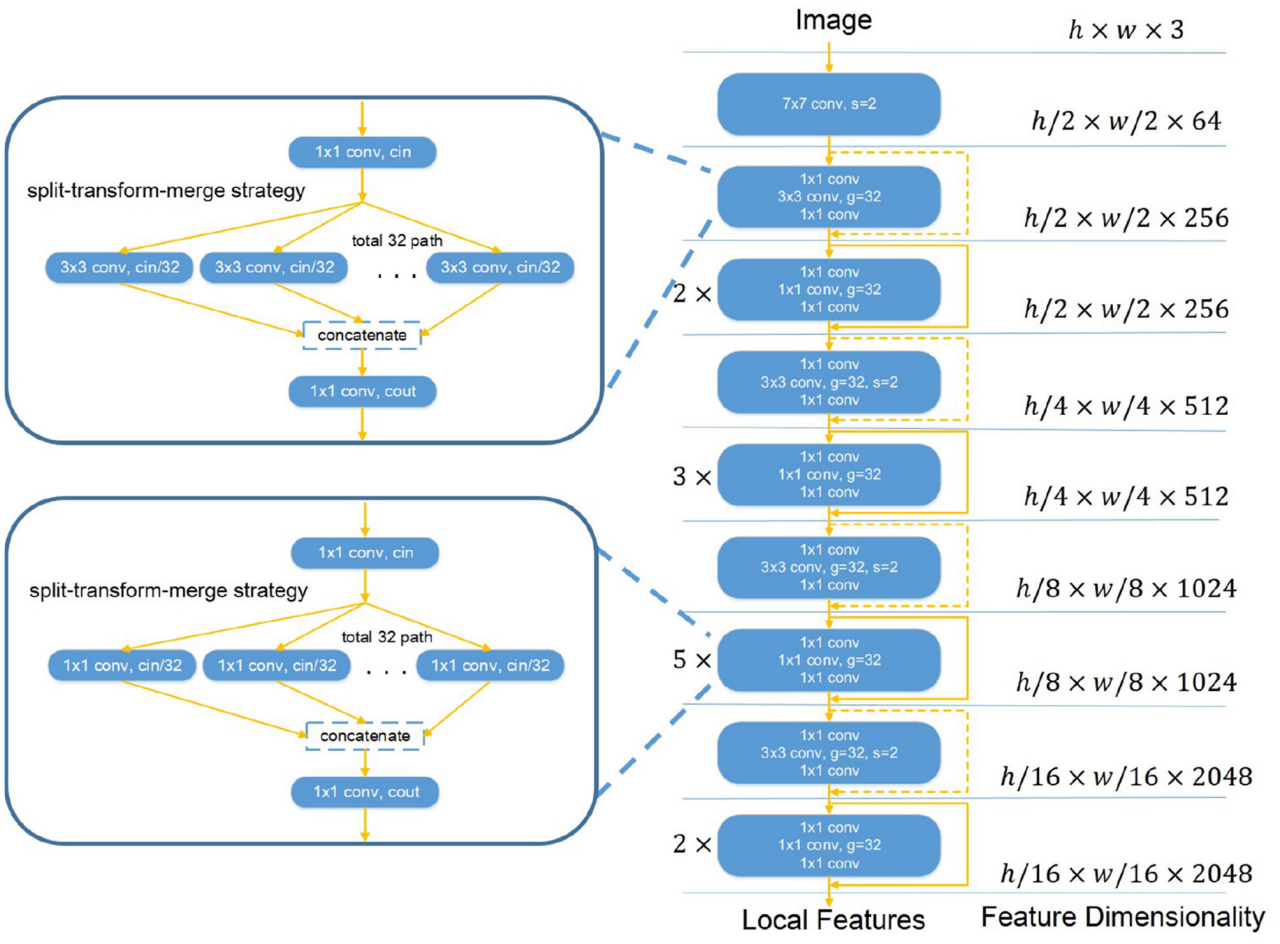
The overall structure of Restricted ResNeXt. Restricted ResNeXt is a variant of ResNeXt architecture. The modification is mainly in the replacement of many 3 × 3 by 1 × 1 convolutions. Moreover, Restricted ResNeXt addresses the gradient vanish problem with the residual learning and reduces the model complexity with the split-transform-merge strategy. 7 × 7 conv denotes the 7 × 7 convolution layer. 1 × 1 conv denotes the 1 × 1 convolution layer. 3 × 3 conv denotes the 3 × 3 convolution layer. s and g denote the stride and group number of the convolution layer, respectively. cin and cout in the split-transform-merge block denote the channel numbers of its convolutions.

We propose a variant of ResNeXt architecture as the backbone given its dominant performance in image analysis (22). Our backbone, Restricted ResNeXt, differs from ResNeXt (22) mainly in the replacement of many 3 × 3 by 1 × 1 convolutions for a restricted receptive field (see Figure 2). Restricted ResNeXt addresses the gradient vanish problem with the residual learning (23) and reduces the model complexity with the split-transform-merge strategy (24). After removing the final classification and pooling layers, an input image with shape *h* × *w* × *c* produces a local feature tensor with shape *h*′ × *w*′ × *c*′. Here, *h*, *w*, and *c* are the height, width, and number of channels of the input image respectively while *h*′ = *h*/16, *w*′ = *w*/16, and *c*′ = 2, 048. The output of this network encodes the images into a set of abstracted feature maps. Each pixel of the feature maps represents a small patch (size 16 × 16) in chest radiographs. The receptive field size of the topmost convolutional layer of Restricted ResNeXt is limited to 39 × 39 pixels. The size of the receptive field can be increased by reducing the number of replaced 3 × 3 convolutions, while the scale of the output remains unchanged.

### 2.2. Position Attention

**Figure 3:**
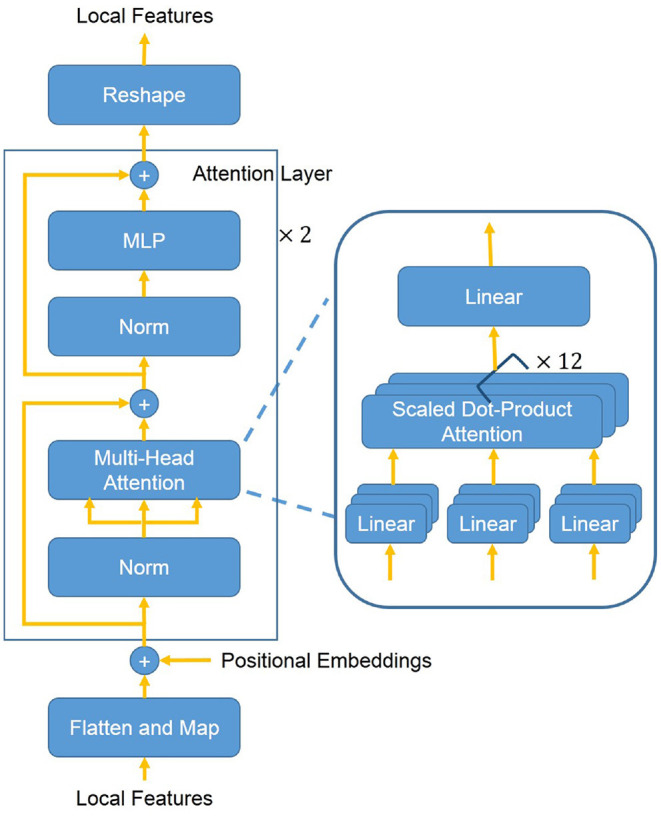
The overall structure of the position attention block. First, the 3D local features are flattened and mapped into a latent *d*-dimensional embedding space. Moreover, learnable positional embeddings are added to the feature embeddings. Then added embeddings are put into a stack of *L* = 2 identical attention layers. The attention layer mainly consists of a MSA mechanism and a MLP. The MSA consists of *M* = 12 concatenated “Scaled Dot-Product Attention”. At last, the flattened features are reshaped back into 3D features.

The position attention block (see Figure 3) highlights local features of the region of interest with Transformer attention mechanism (20). In the position attention block, the local features ***x*** are mapped into a *d*-dimensional (*d* = 1, 024) embeddings ***z***_0_ with position information (Equation 1). The local features ***x*** ∈ ℝ^*h*^′ × *w*′ × *c*′ are reshaped into a sequence of flattened 2D features $x_{p}\in {\mathbb{R}}^{\left(h^{\prime }\cdot w^{\prime }\right)\times c^{\prime }}$. The flattened features ***x***_*p*_ are mapped into a latent *d*-dimensional embedding space using a trainable linear projection. To use position information, learnable positional embeddings (25) are added to the feature embeddings to retain position information as follows:


(1) $$\begin{array}{r} \begin{array}{c} z_{0}=x_{p}\times E+E_{pos}, \end{array} \end{array}$$

where ***E*** ∈ ℝ^*c*^′ × *d* denotes the patch embedding projection and $E_{pos}\in {\mathbb{R}}^{\left(h^{\prime }\cdot w^{\prime }\right)\times d}$ denotes the positional embeddings. Then, *d*-dimensional embeddings ***z***_0_ are put into a stack of *L* = 2 identical attention layers. Each layer has two sub-layers including a multi-head self-attention (MSA) mechanism and a small multi-layer perceptron (MLP) with one hidden layer. The MSA is an extension of “Scaled Dot-Product Attention” (20). We run *M* = 12 “Scaled Dot-Product Attention” operations and project their concatenated outputs in the MSA. We employ a residual connection (23) around each of the two sub-layers, followed by layer normalization (26). Therefore the output features of the *l*-th layer can be written as follows:


(2) $$\begin{array}{c} \begin{array}{c} z_{l}^{\prime }=\text{MSA}(\text{LN}(z_{l-1}))+z_{l-1}, \end{array} \end{array}$$

(3) $$\begin{array}{c} \begin{array}{c} z_{l}=\text{MLP}(\text{LN}(z_{l}^{\prime }))+z_{l}^{\prime }, \end{array} \end{array}$$

where *l* ∈ {1, 2} is the layer number, ***z***_*l*_ denotes the output by the *l*-th layer, and LN denotes the layer normalization operator. At last, the 2D features ***z***_2_ are reshaped back into 3D features ***x***′ ∈ ℝ^*h*^′ × *w*′ × *d*.

### 2.3. Segmentation and Classification

Our model divides the input image into *h*′ × *w*′ patch grid. Each patch is assigned a probability of the diseases by a small FCN (27) with features ***x***′ ∈ ℝ^*h*^′ × *w*′ × *d* as the segmentation result. The small FCN consists of two pointwise convolution layers and sigmoid activation.

Chest L-Transformer is only trained with image-level annotations. To aggregate the pixel-level evidences to an image-level decision, a smooth and convex approximation of the max and average functions (28) is chosen to build Log-Sum-Exp voting as follows:


(4) pk=1rlog1h′×w′∑0<i≤h′0<j≤w′exp(r×pi,jk),

where *p*^*k*^ is the probability of the *k*-th class for an image and $p_{i,j}^{k}$ is the probability of the *k*-th class for the patch at location (*i, j*). *r* is a positive hyper-parameter controlling the smoothness. Log-Sum-Exp voting will be a max function for *r* → ∞ and be an average function for *r* → 0. With *r*, the voting function assigns larger weights to the more important patches.

In chest radiographs, not all the areas are related to thoracic diseases. Although increasing *r* can decrease the weight of these unrelated areas in the voting process, the weight of less important areas of lesions will also be turned to a small value. The model may just focus on the more related areas of the lesions and ignore the less related ones. Moreover, a big value of *r* may lead to an overflow in the calculation. To ignore the unrelated areas, we propose adaptive Log-Sum-Exp voting as follows:


(5) $$\begin{array}{l} p^{k}=\frac{1}{r}\log[\frac{1}{h^{\prime }\times w^{\prime }}\sum_{\begin{array}{c} 0<\;i\leq h^{\prime }\\ 0\;<j\leq w^{\prime } \end{array}}I\times \exp(r\times p_{i,j}^{k})],\\ I=\{\begin{array}{ll} 1, & if\;p_{i,j}^{k}\geq t\times \max_{i,j}(p_{i,j}^{k})\\ 0, & otherwise\; \end{array},\;0\;<t\leq 1. \end{array}$$

We filter the unrelated areas with an adaptive threshold $t\times \underset{i,j}{\max}\left(p_{i,j}^{k}\right)$. The patches with similar evidences will have similar probabilities. With the threshold $t\times \underset{i,j}{\max}\left(p_{i,j}^{k}\right)$, only the patches similar to the most likely abnormal patch participate in the voting. Adaptive Log-Sum-Exp voting adapts the range of voting patches according to their class evidences automatically. *t* controls how similar the voting patches should be to the most likely abnormal patch. Adaptive Log-Sum-Exp voting guarantees only the patches related to diseases involve in the production of image-level probability *p*^*k*^. For the images of diseases, the model will ignore the unrelated areas with this voting function. For the images of normal persons, the model will take more attention to assigning the areas, which are easier to misclassify as lesions, a correct label.

At last, we combine Log-Sum-Exp voting (including adaptive Log-Sum-Exp voting) with the α-balanced focal loss (29) as the weakly supervised loss:


(6) $$\begin{array}{c} \begin{array}{c} L=\sum_{k}[-\alpha y^{k}{\left(1-p^{k}\right)}^{\gamma }\log(p^{k})-(1-\alpha )(1-y^{k}){\left(p^{k}\right)}^{\gamma }\log(1-p^{k})], \end{array} \end{array}$$

where *y*^*k*^ is the binary label of the *k*-th class. The focal loss is initially applied in the object detection task to deal with the foreground-background imbalance. Here, we introduce it to the weakly supervised loss of Chest L-Transformer. Parameter γ is used to down-weight easy cases and focus training on hard-classified cases. Parameter α balances the importance of positive/negative cases.

## 3. Experiments

### 3.1. Datasets

We utilize the SIIM-ACR Pneumothorax Segmentation dataset (30) to verify the proposed method. The dataset contains 12,047 frontal-view chest radiographs with pixel-level annotations, in which 2,669 chest radiographs contain lung pneumothorax and 9,378 chest radiographs have no pneumothorax. The chest radiographs were directly extracted from the DICOM file and resized as 1, 024 × 1, 024 bitmap images. Six board-certified radiologists participated in the annotation process. All annotations were then independently reviewed by 12 thoracic radiologists followed by adjudication by an additional thoracic radiologist.

### 3.2. Metrics

To assess the classification performance of Chest L-Transformer, we compute the area under the receiver operating characteristic curve (AUC), sensitivity, specificity, and F1 score on the testing set. Intersection over union (IoU) is computed to assess the segmentation performance.

Sensitivity and specificity are statistical measures of the performance of a binary classification test. The F1 score is used to measure the test accuracy. AUC is equal to the probability that a classifier will rank a randomly chosen positive instance higher than a randomly chosen negative one.


(7) $$\begin{array}{c} \text{sensitivity}=\frac{\text{TP}}{\text{TP + FN}}, \end{array}$$

(8) $$\begin{array}{c} \text{specificity}=\frac{\text{TN}}{\text{TN + FP}}, \end{array}$$

(9) $$\begin{array}{c} \text{F1}=\frac{\text{2TP}}{\text{2TP + FP + FN}}, \end{array}$$

where true positive, false positive, true negative, and false negative are denoted as TP, FP, TN, and FN, respectively.

IoU, also known as the Jaccard similarity coefficient, is a statistic used for gauging the similarity and diversity of sample sets. IoU can be used to compare the pixel-wise agreement between a predicted segmentation and its corresponding ground truth:


(10) $$\begin{array}{r} J\left(A,B\right)=\frac{\left|A\cap B\right|}{\left|A\cup B\right|}. \end{array}$$

*A* is the predicted set of pixels and *B* is the ground truth.

### 3.3. Experimental Settings

The SIIM-ACR Pneumothorax dataset is used to evaluate the classification and segmentation performance of the proposed Chest L-Transformer with 7:1:2 training:validation:test set split with no intersection. We performed an ablation study to show the effects of different blocks of Chest L-Transformer. First, we train a model with ResNeXt-50 as the backbone without position attention. The second model is Restricted ResNeXt without position attention. The third model is Restricted ResNeXt with position attention. Adaptive Log-Sum-Exp voting is utilized for the three models. The models are named as RNX50-LVT, rRNX50-LVT, and rRNX50-LVT-PA, respectively. Finally, we perform an ablation study for different versions of voting. We compare four voting functions: rRNX50-LVT-PA (adaptive Log-Sum-Exp voting), rRNX50-LV-PA (Log-Sum-Exp voting), rRNX50-AV-PA (average voting), and rRNX50-MV-PA (max voting). As shown in (9), we also train Chest L-Transformer with 400 radiographs with pixel-level annotations and the rest of the dataset with image-level annotations. The binary cross-entropy loss and Dice loss are used for the pixel-level annotated data (4).

The stochastic gradient descent (SGD) optimizer with momentum (0.9) (31) is used to train 500 epochs with an initial learning rate of 0.001. The learning rate is reduced by 0.3 when the training loss stops. We train our model with a batch size of 8 and resize the original images to 512 × 512 as the input. The parameters *r*, *t*, α, and γ are set to 8, 0.6, 0.6, and 2, respectively. In our experiments, we determine them with a search on 10% of the training and validation set. Chest L-Transformer is implemented in Python using PyTorch framework. Referring to the experiment in (3), we initialize the backbones with pre-trained weights.

## 4. Results

### 4.1. Classification

**Figure 4:**
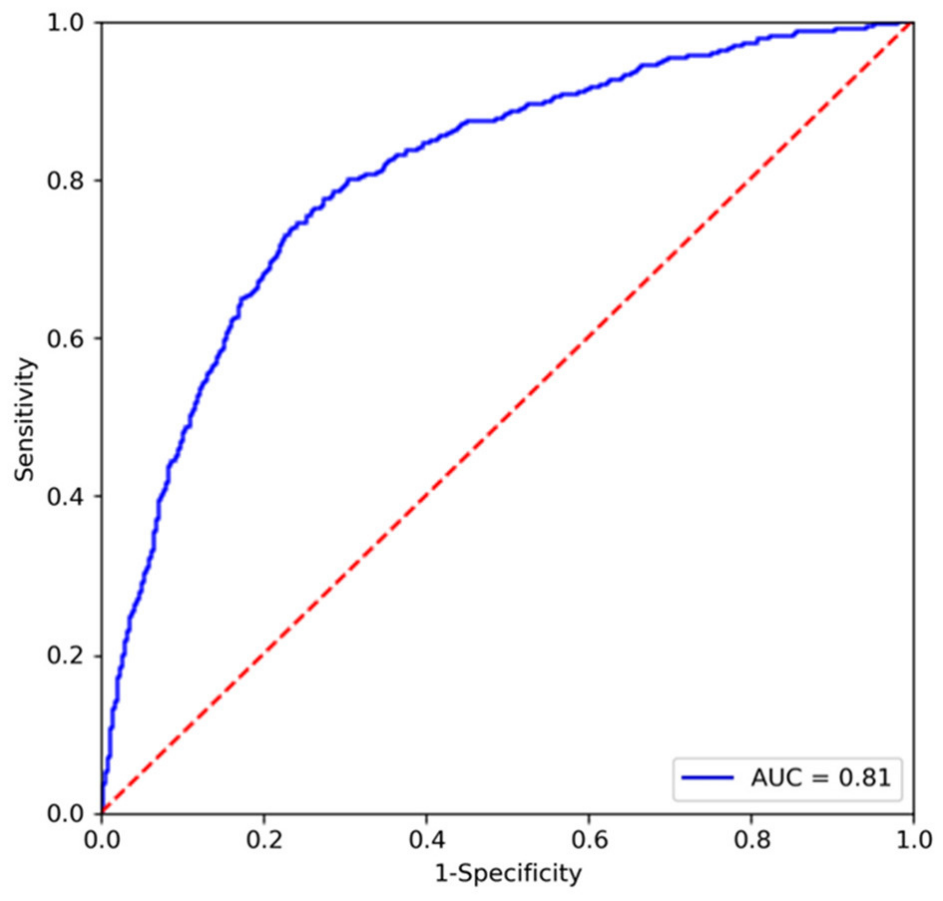
ROC curve of Chest L-Transformer on the testing set.

We conduct an experiment to evaluate the performance on the classification task and compare it to the state-of-the-art segmentation models on the SIIM-ACR Pneumothorax dataset. As few weakly supervised segmentation models on chest radiographs are available, we compare Chest L-Transformer with some supervised models: Mask R-CNN (2, 32) and U-net (2, 3, 33). Chest L-Transformer is trained only with image-level annotations in a weakly supervised manner. The supervised segmentation methods are trained with pixel-level annotations in a supervised manner. We used the maximum probability of lesion areas in a radiograph as the classification probability of supervised segmentation models (2). Moreover, Chest L-Transformer is compared with the classification model ResNeXt (22). The classification performance of Chest L-Transformer is shown in Table 1. Chest L-Transformer achieve an AUC of 0.81, slightly worse than supervised segmentation models (Mask R-CNN AUC = 0.84, U-net AUC = 0.85) and classification model (ResNeXt AUC = 0.84). The receiver operating characteristic (ROC) curve of Chest L-Transformer is illustrated in Figure 4. The results validate the classification effectiveness of Chest L-Transformer.

**Table 1:** Comparison of Chest L-Transformer with the state-of-the-art models (classification).

**Model**	**Main method**	**AUC**	**F1**	**Sensitivity**	**Specificity**
Mask R-CNN	Supervised	0.84	0.60	0.63	0.87
U-net	Supervised	0.85	0.54	0.43	0.85
ResNeXt	Classification	0.84	0.53	0.43	0.95
Chest L-Transformer	Weakly Supervised	0.81	0.57	0.67	0.79

### 4.2. Segmentation

**Figure 5:**
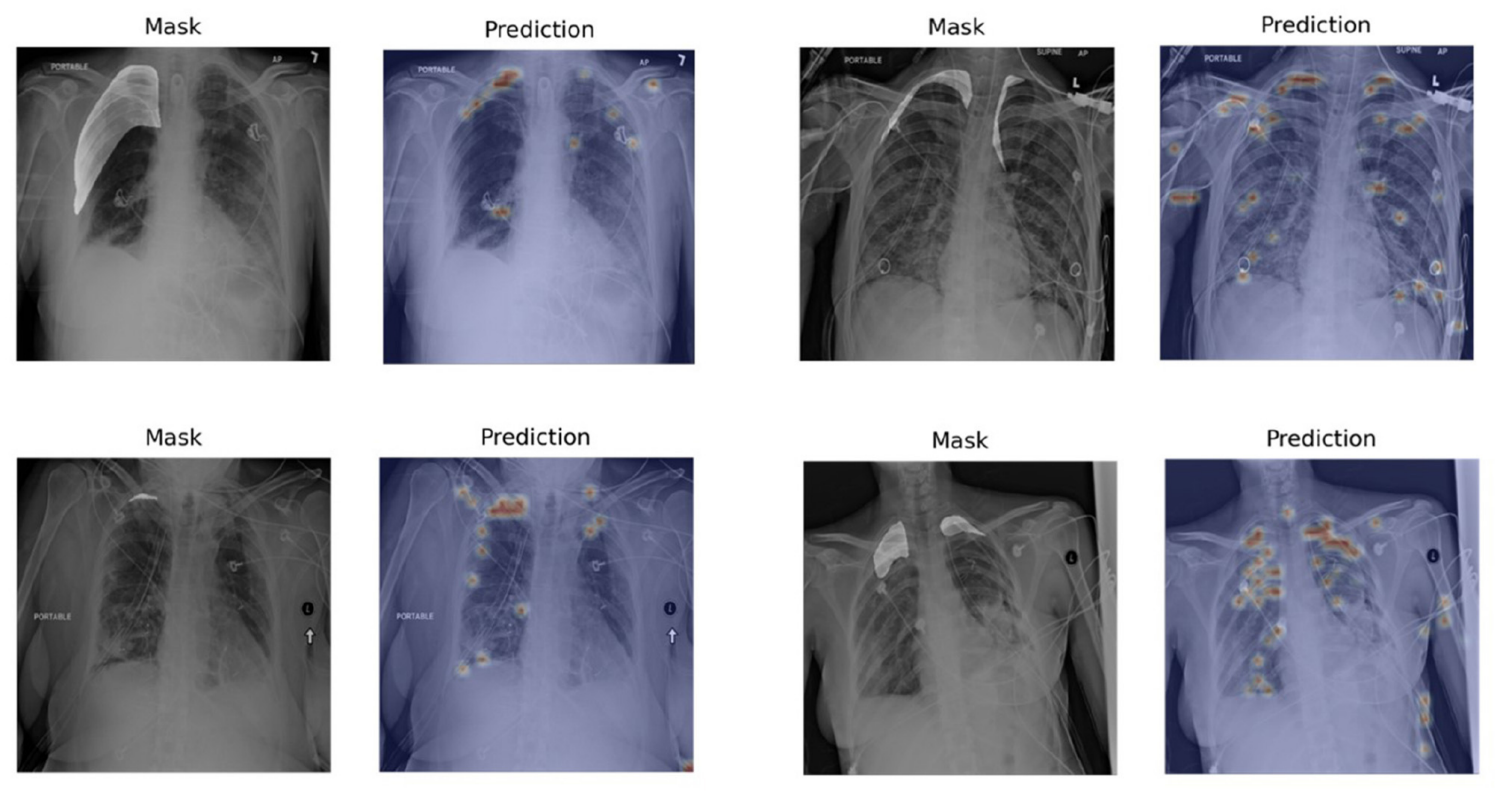
Examples of segmentation visualization on the testing set. The visualization is generated by rendering the pixel-level outputs as heatmaps and overlapping on the original images. The left image in each pair is the original chest radiograph with highlighted masks and the right one is the segmentation visualization.

To evaluate the performance of Chest L-Transformer for segmentation, we computed IoU on the testing set, compared with Mask R-CNN (2, 32), U-net (2, 3, 33), which are trained with pixel-level annotations, and Tiramisu with CNN attention (9), which is trained with image-level annotations, shown in Table 2. Chest L-Transformer achieves an effective result (IoU of 0.70). It performs slightly worse than Mask R-CNN (IoU = 0.75) and U-net (IoU = 0.76) with supervised training. Moreover, Chest L-Transformer outperforms the state-of-the-art weakly supervised model (9) (Tiramisu IoU = 0.13). After added pixel-level annotations, Chest L-Transformer outperforms the state-of-the-art weakly supervised model (9) with IoU increased by 10.4%. Figure 5 shows a few examples of the weakly supervised predictions output by Chest L-Transformer.

**Table 2:** Comparison of Chest L-Transformer with the state-of-the-art segmentation models (segmentation).

**Model**	**Main method**	**IoU**
Mask R-CNN	Supervised	0.75
U-net	Supervised	0.76
Tiramisu	Weakly supervised	0.13
Tiramisu	Weakly supervised + 400 pixel-level annotated radiographs	0.67
Chest L-Transformer	Weakly supervised	0.70
Chest L-Transformer	Weakly supervised + 400 pixel-level annotated radiographs	0.74

“*+ 400 pixel-level annotated radiographs” means that the model is trained with 400 radiographs with pixel-level annotations and the rest of the dataset with image-level annotations*.

### 4.3. Ablation Study

For the ablation study, we study the effectiveness of our modified backbone, position attention block, and proposed voting function.

Table 3 shows the classification results of the ablation study of the architecture of Chest L-Transformer (backbone and position attention block) with the AUC, F1 score, sensitivity, and specificity, while segmentation results of IoU are shown in Table 4. Compared with RNX50-LVT (AUC = 0.80, IoU = 0.62), the classification result of rRNX50-LVT (AUC = 0.74) is worse, but the segmentation result is significantly improved (IoU = 0.69). Although the classification performance decreases, a remarkable improvement in segmentation is achieved by applying Restricted ResNeXt to extract the local features. Compared with rRNX50-LVT, rRNX50-LVT-PA achieves improvements in both classification (AUC = 0.81) and segmentation (IoU = 0.70) with the addition of position attention by 9.5% and 1.4%, respectively. Moreover, rRNX50-LVT-PA outperforms RNX50-LVT in both classification and segmentation.

**Table 3:** Analyzing different architectures of Chest L-Transformer (classification).

**Model**	**AUC**	**F1**	**Sensitivity**	**Specificity**
RNX50-LVT	0.80	0.60	0.62	0.80
rRNX50-LVT	0.74	0.41	0.35	0.90
rRNX50-LVT-PA	**0.81**	0.57	0.67	0.79

*Numbers in bold indicate the best result among the models*.

**Table 4:** Analyzing different architectures of Chest L-Transformer (segmentation).

**Model**	**IoU**
RNX50-LVT	0.62
rRNX50-LVT	0.69
rRNX50-LVT-PA	**0.70**

*Numbers in bold indicate the best result among the models*.

Table 5 shows the classification results of the ablation study of voting functions of Chest L-Transformer with the AUC, F1 score, sensitivity, and specificity, while segmentation results of IoU are shown in Table 6. Among the compared models, rRNX50-MV-PA achieves the worst AUC of 0.66 and IoU of 0.61. rRNX50-AV-PA achieves an AUC of 0.78 and an IoU of 0.66. With Log-Sum-Exp voting, rRNX50-LV-PA (AUC = 0.78, IoU = 0.68) performs better than rRNX50-AV-PA and rRNX50-AV-PA. rRNX50-LVT-PA achieved the best result (AUC = 0.81, IoU = 0.70).

**Table 5:** Analyzing different voting functions of Chest L-Transformer (classification).

**Model**	**AUC**	**F1**	**Sensitivity**	**Specificity**
rRNX50-AV-PA	0.78	0.53	0.55	0.85
rRNX50-MV-PA	0.66	0.38	0.40	0.79
rRNX50-LV-PA	0.78	0.51	0.49	0.88
rRNX50-LVT-PA	**0.81**	0.57	0.67	0.79

*Numbers in bold indicate the best result among the models*.

**Table 6:** Analyzing different voting functions of Chest L-Transformer (segmentation).

**Model**	**IoU**
rRNX50-AV-PA	0.66
rRNX50-MV-PA	0.61
rRNX50-LV-PA	0.68
rRNX50-LVT-PA	**0.70**

*Numbers in bold indicate the best result among the models*.

## 5. Discussion

We propose Chest L-Transformer for the weakly chest radiograph segmentation and classification. Chest L-Transformer is designed with a restricted receptive field backbone to analyze the contribution of each patch to the final image-level decision. Furthermore, Chest L-Transformer focuses on disease prone areas and highlights salient features useful for the diagnostic task by adding Transformer attention mechanism. Log-Sum-Exp voting and its variant are proposed to aggregate the pixel-level evidences to an image-level decision. Chest L-Transformer outperforms the state-of-the-art weakly supervised model and is comparable to the supervised segmentation and classification models (Tables 1, 2).

Extracting features from the whole image makes the pixel assignments difficult (16). The weakly supervised segmentation accuracy is depressed by the misclassification of the symmetrical positions of the lesions (9). Thus, we propose Restricted ResNeXt to extract local features with a simple modification of ResNeXt. Compared with RNX50-LVT, although the classification performance of rRNX50-LVT decreases (Table 3), it achieves remarkable improvement in segmentation (Table 4). Given the simplicity modification, the architecture of Restricted ResNeXt can be easily generalized to other deep learning models to trade a bit of classification accuracy for better weakly supervised segmentation.

The attention mechanism is an effective feature learning technique shown to be helpful in promoting the performances of image analysis models. The diseases often have special disease prone areas. But CNN attention modules treat areas of the whole image equally and fail to model the relationship between the lesions and their position (9–12). To make use of the position information, we introduce Transformer attention mechanism into our model for the position attention block. Learned positional embeddings are added to the feature embeddings to make the position attention block sensitive to certain positions. The prediction ability of Chest L-Transformer is enhanced with additional position attention. This is demonstrated in the comparison of the rRNX50-LVT and rRNX50-LVT-PA (Tables 3, 4). Moreover, the enhanced prediction of Chest L-Transformer outperforms the model with global features, RNX50-LVT (Tables 3, 4). The classification accuracy depressed by local features is offset by position attention. Chest L-Transformer can serve physicians in thoracic disease diagnosis with the effective classification and position information of findings.

To unify classification and segmentation into the same underlying prediction model, we proposed Log-Sum-Exp voting and its variant. In the ablation study, we compare the performance of different voting functions. The average voting used by the previous models achieves high accuracy in classification (Table 5) but low segmentation results (Table 6). It assigns the same weight to all patches of the image in the voting. This may lead to the misclassification of no lesion patches in the abnormal image. The model with the maximum voting is difficult to converge and achieves disappointing results in both classification and segmentation (Tables 5, 6). Log-Sum-Exp voting is proposed to take the place of the two frequently-used functions. It assigns more important patches larger weights than the less important ones. The Log-Sum-Exp voting outperforms these two functions in both classification and segmentation (Tables 5, 6). Chest radiographs contain some patches which are unrelated to the disease. To ignore the unrelated areas, we proposed adaptive Log-Sum-Exp voting, which adapts the range of voting patches with their class evidences automatically. With an adaptive threshold, Chest L-Transformer achieves further improvement in the two prediction tasks (Tables 5, 6).

Chest L-Transformer predicts rough areas of the lesions automatically. The mistakes are mainly led by therapeutic equipment, such as catheters and lines (see Figure 5). Because most of the radiographs with lesions contain therapeutic equipment, this kind of mistake can hardly be avoided with only image-level annotations. Most of the mistakes caused by equipment would be checked out by radiologists quickly. Chest L-Transformer provides good initial areas for the pixel-level annotation and thus reduces the workload of radiologists on this work (30). Chest L-Transformer can speed up the progress of the diagnosis and treatment planning. Moreover, Chest L-Transformer will contribute to the development of medical image data for segmentation, because it reduces the cost of pixel-level annotation.

## 6. Conclusions

In this study, Chest L-Transformer is proposed for weakly supervised segmentation and classification on chest radiographs. The proposed backbone, Restricted ResNeXt, circumvents the misclassification of the symmetrical positions of the lesions. The position attention block embedded into Chest L-Transformer can model the position information and further provide improvement for predictions. Moreover, the Log-Sum-Exp voting and its variant aggregate the pixel-level evidences effectively. We have shown that Chest L-Transformer obtains accurate segmentation and classification predictions with image-level annotations. Therefore, Chest L-Transformer can contribute to the auxiliary diagnosis of thoracic diseases and the development of chest radiograph segmentation datasets. Moreover, the architecture of Chest L-Transformer can be easily generalized to other deep learning models for weakly supervised segmentation.

## Data Availability Statement

The original contributions presented in the study are included in the article/supplementary material, further inquiries can be directed to the corresponding author/s.

## Author Contributions

HG and HW conceived the idea for this study. HW worked on the end-to-end implementation of the study. JW provided relevant insights on the clinical impact of the research work and handled the redaction of the manuscript. PQ managed the project. PQ and JW provided the funding for the research. All authors contributed to the article and approved the submitted version.

## Funding

This work was supported by the National Natural Science Foundation of China (Grant Numbers 61633006 and 81872247), the Fundamental Research Funds for the Central Universities, China (Grant Number DUT21YG118), and “1+X” program for Clinical Competency enhancement-Clinical Research Incubation Project, The Second Hospital of Dalian Medical University (Grant Number 2022JCXKYB07).

## Conflict of Interest

The authors declare that the research was conducted in the absence of any commercial or financial relationships that could be construed as a potential conflict of interest.

## Publisher's Note

All claims expressed in this article are solely those of the authors and do not necessarily represent those of their affiliated organizations, or those of the publisher, the editors and the reviewers. Any product that may be evaluated in this article, or claim that may be made by its manufacturer, is not guaranteed or endorsed by the publisher.
